# Heterotrophic bacteria trigger transcriptome remodelling in the photosynthetic picoeukaryote *Micromonas commoda*

**DOI:** 10.1111/1758-2229.13285

**Published:** 2024-05-22

**Authors:** Maria Hamilton, Frank Xavier Ferrer‐González, Mary Ann Moran

**Affiliations:** ^1^ Department of Marine Sciences University of Georgia Athens Georgia USA; ^2^ School of Oceanography University of Washington Seattle Washington USA

## Abstract

Marine biogeochemical cycles are built on interactions between surface ocean microbes, particularly those connecting phytoplankton primary producers to heterotrophic bacteria. Details of these associations are not well understood, especially in the case of direct influences of bacteria on phytoplankton physiology. Here we catalogue how the presence of three marine bacteria (*Ruegeria pomeroyi* DSS‐3, *Stenotrophomonas* sp. SKA14 and *Polaribacter dokdonensis* MED152) individually and uniquely impact gene expression of the picoeukaryotic alga *Micromonas commoda* RCC 299. We find a dramatic transcriptomic remodelling by *M. commoda* after 8 h in co‐culture, followed by an increase in cell numbers by 56 h compared with the axenic cultures. Some aspects of the algal transcriptomic response are conserved across all three bacterial co‐cultures, including an unexpected reduction in relative expression of photosynthesis and carbon fixation pathways. Expression differences restricted to a single bacterium are also observed, with the Flavobacteriia *P. dokdonensis* uniquely eliciting changes in relative expression of algal genes involved in biotin biosynthesis and the acquisition and assimilation of nitrogen. This study reveals that *M. commoda* has rapid and extensive responses to heterotrophic bacteria in ways that are generalizable, as well as in a taxon specific manner, with implications for the diversity of phytoplankton‐bacteria interactions ongoing in the surface ocean.

## Graphical abstract

The small green alga *Micromonas commoda* altered relative expression of 17% of its genome in co‐cultures with each of three surface ocean bacterial taxa, compared with axenic growth. Transcriptional responses in 377 genes occurred regardless of the bacterium, dominated by changes in photosynthesis, carbon storage and cell division expression. Yet another 1324 responses were unique to one of the bacteria. The number and diversity of effects of marine bacteria on *M. commoda* physiology were unanticipated.

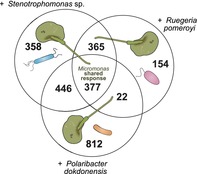

## INTRODUCTION

Marine eukaryotic and prokaryotic phytoplankton are the main source of carbon supporting bacterial heterotrophy in the surface ocean, an interaction that represents one of the largest fluxes of organic carbon on Earth (Moran et al., 2022). Unfortunately, determining the identity and transformation rates of key phytoplankton‐produced metabolites remains a stubborn problem in marine chemistry due both to low metabolite concentrations following efficient uptake by bacteria and their co‐elution with highly concentrated sea salts (Widner et al., 2021). Some classes of metabolites can be studied with targeted chemical approaches (Longnecker et al., 2024) but ultimately these provide a biased view of the complete metabolite pool. Advancements in transcriptomics (Ferrer‐González et al., 2021; Landa et al., 2017; McCarren et al., 2010; Sharma et al., 2014) bring in insights from gene and protein expression, helping to side‐step analytical chemistry challenges (Moran et al., 2022) and gain a better understanding of small molecule flux between surface ocean phytoplankton and bacteria.

Metabolite flow during microbial interactions can be a reciprocal relationship. In one of the most quantitatively important interactions, marine bacteria convert the organic compounds present in phytoplankton exometabolites back into their inorganic components, with the remineralised nutrients subsequently used by phytoplankton to fuel new primary production. This relationship has the added complexity, however, of subsequent competition between phytoplankton and bacteria for the newly available nutrients (Bratbak & Thingstad, 1986; Calfee et al., 2022). Complex reciprocal interactions are also evident in microbial vitamin exchange (Cooper et al., 2019; Croft et al., 2005; Kazamia et al., 2012), antagonistic relationships (Findlay & Patil, 1984; Segev et al., 2016; Seyedsayamdost et al., 2011) and chemotaxis and attachment (Kogure et al., 1981; Mayali et al., 2011; Stocker & Seymour, 2012). Characterisation of these molecular interactions, despite methodological challenges, is crucial to building understanding of the microbial roles in the regulation of global biogeochemical cycles (Cole, 1982; Seymour et al., 2017).

A recent surge in research on surface ocean microbial interactions has focused heavily on the bacterial side of the relationships, in part because of the relative ease with which bacterial genomes can be sequenced, manipulated and functionally annotated compared with those of eukaryotic phytoplankton (Sibbald & Archibald, 2017). Thus a substantial gap exists in knowledge of eukaryotic phytoplankton molecular level responses to the presence of heterotrophic bacteria. Of the currently available phytoplankton ‘omics data, most are focused on two diatoms: *Pseudo‐nitzschia* multiseries (Amin et al., 2015) and *Thalassiosira pseudonana* (Bartolek et al., 2022; Durham et al., 2017). Both have sequenced genomes (Armbrust et al., 2004, 2011) and *T. pseudonana* is also a model organism for marine algal genetics (Bowler et al., 2010; Poulsen et al., 2006). Moreover, the contributions of diatoms to global biogeochemical processes are substantial (Armbrust, 2009) and warrant attention. Under future ocean conditions, however, the abundance of larger sized phytoplankton is likely to wane and smaller‐sized taxa are predicted to increase in biogeochemical impact as the ocean warms and oligotrophic waters expand (Polovina et al., 2008). Picoplankton (<2–3 μm diameter) are among the phytoplankton taxa predicted to be climate change ‘winners’ (Morán et al., 2010; Winder & Sommer, 2012). These include members of the green algal genus *Micromonas*, which has a wide thermal niche and an oligotrophy‐adapted nutrient acquisition strategy conferred by its size (Demory et al., 2019; Li et al., 2009). Predicted future importance in combination with the availability of sequenced genomes make *Micromonas* a prime candidate for investigations into phytoplankton‐bacterial interactions relevant in a changing climate.

Our previous work established individual co‐cultures between *Micromonas commoda* and three heterotrophic bacterial taxa: *Ruegeria pomeroyi* DSS‐3, *Stenotrophomonas* sp. SKA14 and *Polaribacter dokdonensis* MED152 (Ferrer‐González et al., 2023). Gene expression by the bacteria was the focus of the previous study, using transcription patterns as biological reporters of metabolites released by the phytoplankter. In this study, we used the same system to instead leverage phytoplankter gene expression as a reporter of its physiological and ecological interface with the bacteria. Gene expression by *M. commoda* grown in co‐culture with each bacterium individually was compared with expression under axenic conditions, and the types and diversity of responses initiated by the phytoplankter in the presence of heterotrophic bacteria were investigated.

## EXPERIMENTAL PROCEDURES

### Experimental setup

Axenic *Micromonas commoda* RCC299 (National Center for Marine Algae, NMCA) was inoculated into 1 L of organic carbon‐free L1‐Si medium (salinity = 35) containing replete concentrations of nitrogen (880 μM NaNO_3_), phosphorus (36 μM NaH_2_PO_4_), vitamins and trace metals (Table S1). Cultures were established in 1.9 L vented polystyrene tissue culture flasks kept at 18°C in 160 μmol photons m^−2^ s^−1^ on a 16:8 h light: dark cycle. Three marine bacterial strains, *R. pomeroyi* DSS‐3, *Stenotrophomonas* sp. SKA14 and *P. dokdonensis* MED152 were pre‐grown overnight in YTSS medium. Bacteria were washed five times in sterile L1 medium before inoculation individually at ~10^6^ cells ml^−1^ into *M. commoda* cultures grown for 7 d (*n* = 4 per bacterial strain). Axenic phytoplankton cultures were included as a control. All three co‐culture treatments and the axenic treatment were established with 8 replicate flasks, with four harvested at 8 h and four at 56 h. At the 8 h time point, 500 mL of co‐culture and axenic culture from four replicates of each treatment were filtered through 0.2 μm pore‐size 47 mm Supor filters to capture *M. commoda* and bacterial cells, flash frozen and stored at −80°C. An additional 50 mL was similarly filtered, and the spent medium frozen and stored at −20°C for nutrient analyses. The remaining four replicates were maintained in the light for 56 h before harvesting. Samples for flow cytometry were taken at 0, 8 and 56 h, fixed at a final concentration of 1% glutaraldehyde, incubated at 4°C for 20 min and stored at −80°C.

### RNA extraction and transcriptome sequencing

The RNA extraction and sequencing was performed as described in Ferrer‐González et al. (2023). In brief, filters (*n* = 4 or 3 per treatment) were individually incubated in TE buffer, SDS (0.6% final concentration) and proteinase K (120 ng μL^−1^ final concentration), then extracted in equal volumes of acid phenol:chloroform:isoamyl alcohol (25:24:1) and chloroform: isoamyl alcohol (24:1). After centrifugation, the supernatant was mixed with 1 volume of isopropanol and sheared by passage through a 21 g syringe needle. The samples were then incubated overnight at −20°C, centrifuged again, and the pellet was resuspended in RNAase‐free water. The Turbo DNA‐free kit (Invitrogen, Waltham, MA, USA) was used to remove DNA, and PCR was performed to check for residual DNA using the 27F/1492R primer set targeting the 16S rRNA gene (temperature programme: 30 s at 98°C, 35 cycles of 30 s at 95°C, 30 s at 50°C and 60 s at 72°C, followed by 15 min at 72°C).

Prior to sequencing, the DNA was processed with the NEBNext rRNA Depletion Kit (E7860; New England Bio Labs, Ipswich, MA), modified to remove *M. commoda* and bacterial rRNA using a custom pool of 160 oligonucleotide probes (Ferrer‐González et al., 2023). The NEBNext Ultra II Directional Kit (E7765) was used for library preparation, which were sequenced at the Georgia Genomics and Bioinformatics Core (Athens, GA, USA) on the NextSeq 2000 platform (SE100; Illumina, San Diego, CA, USA). The average number of reads per sample was 54,176,243 (*n* = 13).

### Differential expression analysis

For the *M. commoda* RCC299 genome, gene model sets and functional annotations were obtained from the most recent assembly (van Baren et al., 2016) through the JGI PhycoCosm genome browser (Grigoriev et al., 2021). Transcripts were mapped to the microbial genomes using the FASTX toolkit, imposing a minimum quality score of 20 over 80% of read length. Reads aligning to an in‐house rRNA database (https://doi.org/10.5281/zenodo.6812122) were removed (SortMeRNA 2.1‐GCCcore‐8.3.0). Remaining reads were mapped to the genomes of *M. commoda* and the heterotrophic bacteria *R. pomeroyi* DSS‐3, *Stenotrophomonas* sp. SKA14 and *P. dokdonensis* MED152 (Bowtie 2) and counted (HTSeq) (Anders et al., 2015; Langmead & Salzberg, 2012) (NCBI RefSeq accession numbers ASM1196v2, ASM15857v1 and ASM15294v2, respectively). *M. commoda* genes with differential expression in the bacterial co‐culture treatments compared with the axenic cultures were identified using DESeq2 (Love et al., 2014) with significance requiring *p* ≤ 0.01 after adjusting for multiple comparisons (padj), and a fold‐difference of at least 2.

Downstream analyses focused on genes with manually curated annotations. This included genes with ‘User Annotations’ in the JGI PhycoCosm genome browser based on previous curation work (van Baren et al., 2016; Worden et al., 2009), as well as new curations. The new manually curated annotations and pathway designations were derived from automated KOG (Eukaryotic Orthologous Groups of proteins) (Koonin et al., 2004) and KEGG (Kyoto Encyclopedia of Genes and Genomes) orthology (Kanehisa, Sato, Kawashima, et al., 2016) classifications. Auxin signalling pathways were annotated based on orthologs identified for *M. commoda* in De Smet et al. (2011). In total, annotations were expanded for the pentose phosphate pathway, glycolysis/gluconeogenesis, TCA and glyoxylate cycles, Calvin cycle, interaction/signalling related genes, nitrogen acquisition and metabolism, vitamin metabolism and amino acid metabolism (Table S2).

### Flow cytometry

An internal standard of 5‐μm fluorescent particles (ACFP‐50‐5; Spherotech, Lake Forest, IL, USA) was added to thawed samples just prior to analysis. Samples were stained with SYBR Green I (final concentration 0.75X; Life Technologies, Waltham, MA, USA) and analysed on an Agilent Quanteon flow cytometer (Acea, Biosciences Inc, San Diego CA) with a 405 nm laser using a 530/30 bandpass filter for SYBR Green (bacteria) and a 695/40 bandpass filter for chlorophyll *a* (phytoplankton).

### Nutrient analysis

Nutrient analyses were performed by the University of Georgia Laboratory of Environmental Analysis. Concentrations of nitrate (NO_3_^−^), nitrite (NO_2_^−^) and phosphate (PO_4_^3−^) were measured using ion chromatography on a DX500 Ion Chromatograph (Dionex Co.) with an initial cartridge treatment (OnGuard‐Ag cartridge from Dionex) performed to remove chloride ions. Measurements for ammonium (NH_4_^+^) were done separately via the phenate method (Clesceri et al., 1998) with spectrophotometric analysis on a Model Spectronic 21D (Spectronic Instrumentation).

### Bacterial functional prediction

To assess the functional differences between the three bacteria based on genomic prediction, KO assignments for genes within each bacterial genome were generated via BlastKOALA (Kanehisa, Sato, & Morishima, 2016). These assignments were input into the KEGG‐Decoder tool (Graham et al., 2018) to determine completeness of metabolic pathways.

## RESULTS

**FIGURE 1:**
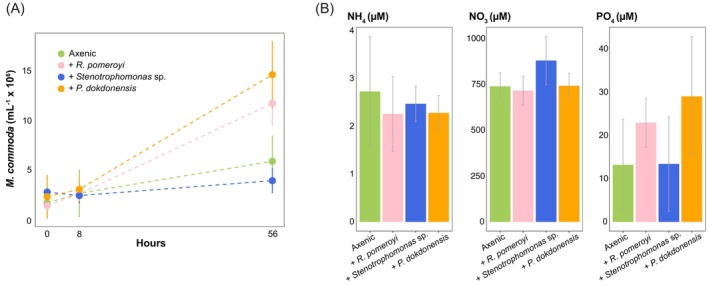
(A) Cell abundances of *Micromonas commoda* based on flow cytometric analysis for co‐cultures and axenic cultures. (B) Inorganic nutrient measurements in spent media of experimental cultures at the 8 h time point. Average values for all data are shown with error bars representing the standard deviation of biological replicates, *n* = 4.

*M. commoda* cell numbers in bacterial co‐cultures were compared with axenic conditions. After 56 h, *M. commoda* exhibited an eight‐fold and six‐fold increase in abundance when in co‐culture with *R. pomeroyi* and *P. dokdonensis*, respectively, compared with a three‐fold increase in the axenic culture (*t*‐test; *p* = 0.014 with *R. pomeroyi*, *p* = 0.006 with *P. dokdonensis*) (Figure 1A). *Stenotrophomonas* sp. did not elicit an increase in *M. commoda* abundance over the axenic cultures.

At the 8 h time point, *M. commoda* already manifested significant transcriptional shifts in the bacterial co‐cultures compared with axenic growth, with bacterial presence inducing differential expression of 9%–16% of the total genes in the *M. commoda* genome. The magnitude of transcriptional responses of *M. commoda* to the bacteria, however, did not correspond to the magnitude of the growth effect at 56 h. *R. pomeroyi* induced the fewest number of differentially expressed genes (923), yet had the largest positive effect on the phytoplankter's growth. The other two bacteria induced higher numbers of differentially expressed genes (1665 for *P. dokdonensis*, 1547 for *Stenotrophomonas* sp.), but only *P. dokdonensis* impacted the growth of *M. commoda*. Nearly 32% (812) of the differentially expressed algal genes identified were unique to co‐cultures with *P. dokdonensis*, compared with 6% (154) and 14% (358) unique genes in the *R. pomeroyi* and *Stenotrophomonas* sp. co‐cultures, respectively. The remainder of the differentially expressed genes were shared between two co‐cultures (468) or by all three (377). Transcriptome analysis therefore revealed a diversity of expression responses by *M. commoda* to co‐cultured bacteria while phenotype analysis revealed a growth rate response.

### *Shared* M. commoda *transcriptional responses indicate changes in photosynthesis and carbon storage*

**FIGURE 2:**
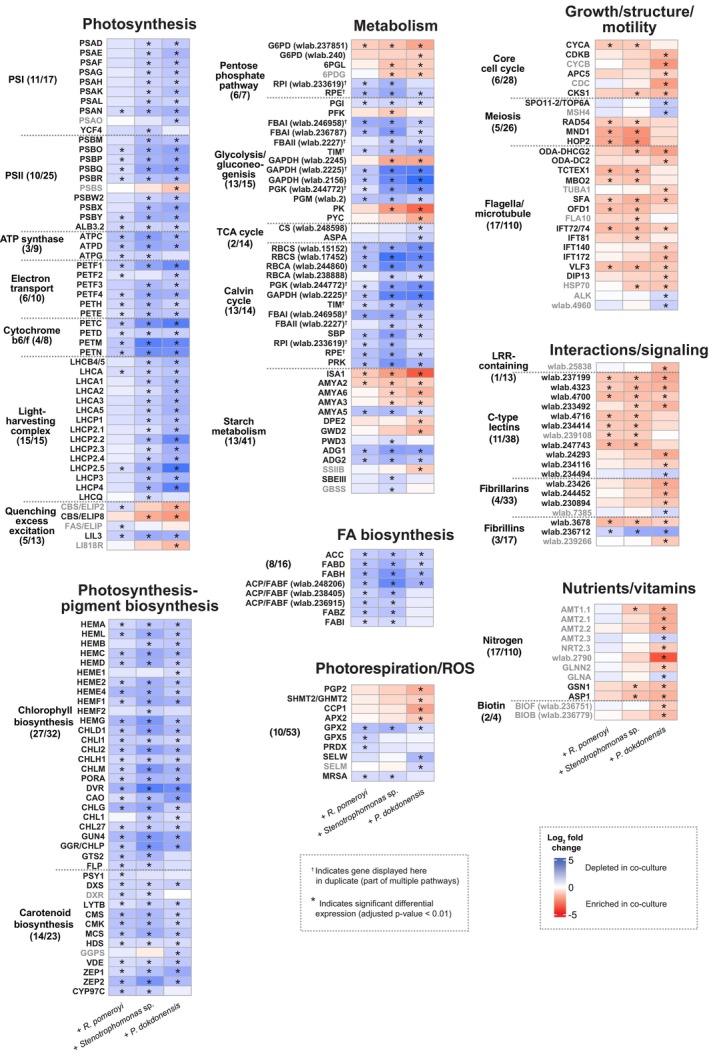
*Micromonas commoda* genes differentially expressed between the axenic and co‐culture treatments. Genes displayed have putative annotations in key pathways. The log_2_ fold change of each gene is indicated by the colour gradient, with blue representing transcript depletion and red representing transcript enrichment in the co‐cultures compared with the axenic cultures. Genes with asterisks had statistically significant differential expression (DESeq2, adjusted *p*‐value <0.01, *n* = 3 or 4). Gene names in black font represent those with a shared directionality of log_2_ fold change within the row (either positive or negative) for all co‐culture treatments. The number of significantly differentially expressed genes out of the total genes in the *M. commoda* genome annotated as part of a pathway is indicated by the numbers on the left‐hand side for each pathway cluster.

As the bacterial species in this study represent three distinct taxonomic lineages that vary in their functional capabilities, we were interested in *M. commoda* expression responses common to all three. One of the most striking shared responses was a reduction by *M. commoda* of relative gene expression for both light‐dependent photosynthetic reactions and carbon fixation in the presence of each bacterium. All major components of the light‐dependent photosynthetic process exhibited this pattern, including photosystem I (PSI), photosystem II (PSII), ATP synthase, electron transport, cytochrome b6/f, light harvesting complex proteins and biosynthesis of photosynthetic pigments (Figure 2), with 89 differentially expressed genes out of 139 total in the combined pathways. Consistent with the depletion in genes involved in the light dependent reactions of photosynthesis, *M. commoda* Calvin cycle transcripts were also depleted in co‐cultures (Figure 2). Together these constituted a puzzling decrease in investment in photosynthetic fixation of CO_2_ while experiencing culture conditions that promoted increased growth. Additional shared transcriptional changes in core metabolic functions included the pentose phosphate pathway and glycolysis/gluconeogenesis genes, although these did not display trends as consistent as the photosynthesis and Calvin cycle pathways (Figure 2).

We also observed reductions in the expression of *M. commoda* genes related to starch accumulation and fatty acid biosynthesis when in co‐culture with all three bacteria. Increases in relative expression were found for two genes encoding starch breakdown (AMYA2 and ISA1) while decreases were found for one gene encoding starch breakdown (AMYA5) and two encoding biosynthesis (ADG1 and ADG2, subunits of ADP‐glucose pyrophosphorylase) (Figure 2). Four genes in the fatty acid biosynthesis pathway were also significantly depleted in all three co‐cultures, including the acetyl‐CoA carboxylase gene encoding the first step in transformation of photosynthesis derived acetyl‐CoA into storage fatty acids.

### *Shared* M. commoda *transcriptional responses indicate changes in cell division, structure and motility*

Similarities in *M. commoda* gene expression responses regardless of bacterial partner were also evident for cell division genes, including those putatively encoding cyclins and cyclin‐dependent kinases (Figure 2). For *R. pomeroyi* and *Stenotrophomonas* sp. co‐cultures, the CYCA gene encoding an A‐type cyclin, likely the main activator of CDKA and thus of early events in mitosis in *Chlamydomonas* (Atkins & Cross, 2018), was significantly enriched in *M. commoda* co‐cultures compared with axenic cultures (Figure 2). For *P. dokdonensis* co‐cultures, the primary inducer of mitosis, the CYCB‐CDKB complex, was enriched and there was a similar pattern of enriched genes either related to (i.e., CDC) or directly encoding (APC5) the anaphase promoting complex, the key regulator of a later phase of mitosis. Despite differences in the precise stage of mitosis induced in *M. commoda*, all three bacteria appeared to trigger cell division, or at least alter the phytoplankter's cell cycle, after 8 h in co‐culture.

Enriched expression in a number of genes involved in structural components, including those annotated for formation and maintenance of flagella (Figure 2) and those assigned to the ‘cytoskeleton’ KOG category (Figure S1), provide further evidence that cell division may have been triggered by the bacteria. Also of note is the observation of enriched expression in one or more of the co‐culture treatments of genes predicted to be specifically involved in flagella beating, including ODA‐DHCG2, ODA‐DC2, TCEX1 and MBO2 (Figure 2). It is unclear if this is indicative of increased motility for *M. commoda* in the presence of bacteria, or simply another copy of a required component as a new flagellum is manufactured during cell division.

### *Shared* M. commoda *transcriptional responses indicate recognition and signalling*

All three bacterial co‐cultures induced a number of putative C‐type lectins, carbohydrate‐binding proteins known to be particularly important for symbiont and pathogen detection in plants and metazoans (Bellande et al., 2017; Wood‐Charlson et al., 2006). One putative fibrillin, typically co‐located with lipid plastoglobules in photosynthetic organisms, was also induced (Figure 2). A substantial number of *M. commoda* genes having the broad KOG class annotation of ‘RNA processing and modification’ had enriched expression in co‐culture with bacteria (Figure S1). While RNA modification is not specific to cell‐to‐cell interactions, it may have supported a molecular mechanism underlying the phytoplankter's response. Many genes broadly annotated as ‘Posttranslational modification, protein turnover, chaperones’ were depleted in expression (Figure S1), again indicating that all three bacterial species likely induced a shift in the phytoplankter's metabolism. A putative leucine‐rich repeat (LRR) gene of unknown function (wlab.25838) was enriched, but only in co‐culture with *P. dokdonensis* (Figure 2). LRR proteins are typically positioned either at the cell surface or in the cytoplasm and, in plants, play roles in immunity and development (Diévart & Clark, 2004; Yue et al., 2012).

### *The* M. commoda *transcriptome reveals a unique response to* P. dokdonensis

Of the three heterotrophic bacteria, *P. dokdonensis* induced a particularly unique transcriptional response in *M. commoda*. The distinctive genes were primarily related to vitamin and nutrient biosynthesis and acquisition, with some abiotic stress‐linked photosynthesis genes showing a unique response as well (Figure 2). Additionally, we found a curious pattern among genes annotated as meiosis associated. Meiotic recombination has not yet been directly observed in *Micromonas*, but genomic analysis uncovered multiple lines of evidence in support of a sexual life‐cycle phase (Worden et al., 2009). Two putative meiosis related genes, SPO11‐2, which forms the double strand DNA breaks needed for meiotic recombination, and MSH4, which promotes cross‐over formation, were significantly depleted in co‐cultures with *P. dokdonensis* compared with axenic cultures (Figure 2). While many meiosis related genes are also important for general DNA repair, these two are specifically expressed during meiosis in plants (Higgins et al., 2004).

Other components of the unique response to *P. dokdonensis* include the significant enrichment in the *M. commoda* transcriptome of genes putatively involved in biotin (vitamin B7) biosynthesis, in nitrogen acquisition and assimilation, and in the algal stress response and non‐photochemical quenching (NPQ) (Figure 2). In *P. dokdonensis* co‐cultures, genes encoding the PSBS component of PSII, two early light‐inducible proteins and the light‐harvesting complex‐like protein LI818, all had significantly enriched expression compared with axenic *M. commoda*. These genes have been previously observed to exhibit increased expression in algae and land plants under conditions of light or nutrient stress (Tzvetkova‐Chevolleau et al., 2007; Ware et al., 2015; Zhu & Green, 2010). The increased expression of transporters for ammonium, nitrate and urea, as well as the activation of the GS‐GOGAT system that we observed for *M. commoda* in co‐culture with *P. dokdonensis* (Figure 2) are also potentially indicative of a nutrient stress signal.

## DISCUSSION

The six‐ and eight‐fold increases in *M. commoda* abundance after 56 h in co‐culture provided initial evidence of physiologically relevant interactions with the bacteria. Previous work has underscored the highly specific growth effects that bacterial taxa can have on phytoplankton (Deng et al., 2022; Le Reun et al., 2023), and here we found growth enhancement of *M. commoda* for two of the three bacteria. The growth benefit to *M. commoda* was not likely attributable to exchange of essential vitamins or trace metals, as these were provided in excess in the medium. Further, inorganic nitrogen and phosphorous measured in the spent media at the 8 h time point remained well above limiting concentrations and were comparable between co‐cultures and axenic control cultures (Figure 1B). Another potential mechanism underlying enhanced cell numbers is bacterial release of a growth altering factor as has been observed in studies with other phytoplankton (Amin et al., 2015; Seyedsayamdost et al., 2011) as well as in land plants (Spaepen & Vanderleyden, 2011). The hormone indole‐3‐acetic‐acid (IAA) has been documented to encourage phytoplankton cell division (Amin et al., 2015), but none of the bacteria in this study have a complete tryptophan‐dependent IAA biosynthesis pathway (Ferrer‐González et al., 2023). While IAA‐based interactions can likely be ruled out, the potential for a growth enhancing metabolite released by the bacteria remains an open question.

**FIGURE 3:**
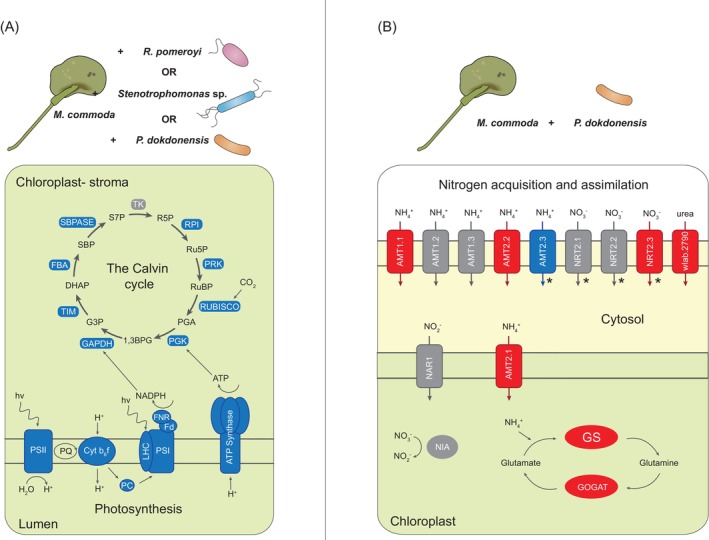
Examples of shared and unique *Micromonas commoda* transcriptional responses to the presence of heterotrophic bacteria. (A) Shared response to all three bacteria in expression of light harvesting reactions of photosynthesis and the Calvin cycle. (B) Unique response to *Polaribacter dokdonensis* in expression of nitrogen acquisition and assimilation. Functional components represented in red indicate enriched expression in co‐cultures and in blue indicate depleted expression. Components represented in grey showed no differential expression. Transporter localization within the *M. commoda* cell depicted in (B) is based on information from McDonald et al. (2010), with asterisks indicating inconsistencies between annotation tools used in prediction.

Transcriptome analysis identified 377 *M. commoda* expression responses that were shared across all three bacterial species. Among these, the most surprising was also the most pronounced: depleted expression of the majority of photosynthesis and carbon fixation genes in co‐cultured *M. commoda* (Figure 3A). This has not been shown previously with marine phytoplankton transcriptomics, and the ecological logic underpinning it is particularly unclear in light of the increased phytoplankton growth rate observed at 56 h. Because the transcriptomic data are compositional, one explanation for a lower proportion of photosynthesis related transcripts could simply be increases in transcripts mediating other pathways. This seems unlikely, however, because the high proportion of photosynthesis transcripts (almost 10% of mapped reads in the axenic cultures) lessens their sensitivity to expression changes in other pathways.

The most probable conclusion is that *M. commoda* indeed shifted transcriptional investment away from capturing light energy and generating sugars from CO_2_ after 8 h in co‐culture with heterotrophic bacteria. Previous studies have shown that marine phytoplankton downregulate photosynthesis genes in response to nitrogen stress (Bender et al., 2014; Jian et al., 2017; Miller et al., 2010; Sun et al., 2013) and light availability (Cuvelier et al., 2017; Diaz et al., 2023; Nymark et al., 2009). Neither of these conditions differed between treatments in our experimental system, however. Alternatively, the decrease in photosynthetic gene expression measured at 8 h could be a transient condition that was not manifested in the form of impaired growth at 56 h (Figure 1A). Phagotrophic mixotrophy, a strategy identified in an increasing number of previously presumed strict phototrophs (Millette et al., 2023), was also considered, but there is no convincing evidence of this trophic mode in the *M. commoda* genome (Jimenez et al., 2021) or in feeding experiments with closely related *Micromonas polaris* (Jimenez et al., 2021; Wilken et al., 2019). A final hypothesis is that the decrease (potentially temporary) of *M. commoda* photosynthesis and carbon fixation expression is linked to osmotrophic uptake of bacterial exudates, lessening the need for *M. commoda* to fix carbon. Other phytoplankton have been shown to use dissolved organic compounds as growth substrates (Balch et al., 2023; Muñoz‐Marín et al., 2013; Yelton et al., 2016), but osmotrophy has not yet been studied in *Micromonas*. While there is scant experimental information on organic substrate transporter genes in any green alga at this point, this question is worth pursuing in future research.

Learning the chemical signals that regulate microbial communication will be key to understanding shared phytoplankton‐bacteria interactions. Chemical signals that have emerged previously include IAA (Amin et al., 2015), DMSP (Barak‐Gavish et al., 2023; Segev et al., 2016), benzoate (Barak‐Gavish et al., 2023) and bacterial quorum sensing molecules (Dow, 2021), among others. The substantial number of *M. commoda* genes having shared expression responses across all three co‐cultures, despite substantial phylogenetic and ecological distinctions among the heterotrophic bacteria (Figure S2), allows for the possibility of a common bacterial signalling molecule that broadly regulates phytoplankton genes. Transcriptional enrichment of CDPK, MAPK and LRR genes was previously found for a marine diatom when in co‐culture with *R. pomeroyi* (Durham et al., 2017), echoing a response that occurs during plant recognition of bacteria (Diévart & Clark, 2004; Ligterink & Hirt, 2001). Here, only one putative LRR‐containing gene was enriched, and only in the *P. dokdonensis* co‐culture (Figure 2). The induced C‐type lectins (Figure 2) are perhaps more likely to be involved in shared responses based on previous studies of interactions between marine metazoans and microbes (Balzano et al., 2015; Wood‐Charlson et al., 2006). These proteins recognise and bind specific glycans, including those found in bacterial lipopolysaccharide, peptidoglycan and capsular material. Fibrillin genes have not yet been studied in eukaryotic algae but multiple lines of evidence, including gene knockout experiments, suggest they are involved in plant immune response to bacteria (Cooper et al., 2003; Kim & Kim, 2022; Singh et al., 2010).

Transcriptome analysis identified 1324 *M. commoda* expression responses that were restricted to a single bacterial species, of which 812 were unique to *P. dokdonensis* co‐cultures. Although the culture medium was amended with biotin, *M. commoda* nonetheless increased investments in de novo biotin synthesis in the presence of *P. dokdonensis*, which is also the only bacterium of the three that requires exogenous biotin (Figure 2). Enriched expression of multiple *M. commoda* nitrogen transporter genes was also evident only in the *P. dokdonensis* co‐cultures (Figure 3B). Past studies with *M. commoda* have shown increases in ammonium and nitrate transporter expression in response to nitrogen stress (McDonald et al., 2010), but here inorganic nitrogen measurements at the time of RNA sampling showed levels well above limitation (Figure 1B) and therefore argue that expression changes were not likely a direct response to drawdown by *P. dokdonensis*. Additional transcriptome evidence that the phytoplankter perceived nitrogen stress included increased expression of the GS‐GOGAT system and photosynthesis related genes known to indicate a general stress response, such as PSBS and LI818R (Ware et al., 2015; Zhu & Green, 2010) (Figures 2 and 3B). *P. dokdonensis* can use only ammonium as its inorganic nitrogen source (González et al., 2008) and is unable to grow with most nitrogen‐containing algal metabolites, including taurine and polyamines (González et al., 2008). Thus, *M. commoda* responses indicative of biotin and nitrogen limitation occurred only during co‐culture with the bacterial species reliant on exogenous biotin and ammonium, and occurred despite replete levels of both molecules in the culture medium. Such specific responses to an individual bacterium without an obvious external signal are indicative of highly sophisticated regulatory controls over phytoplankton‐bacteria interactions.

### Concluding remarks

Members of the eukaryotic picoplankton, including *M. commoda* and its relatives, are among the least studied of the major marine phytoplankton groups, lagging behind in accumulated knowledge of their metabolism, ecology and gene function (Massana, 2011; Worden et al., 2004). Yet, eukaryotic picoplankton and *Micromonas* specifically, are globally important drivers of elemental cycles and distributed widely across ocean biomes, including polar regions where they replace the functional roles of cyanobacteria (Lovejoy et al., 2006). Eukaryotic picoplankton have been predicted to emerge as strong competitors under future ocean conditions (Morán et al., 2010; Winder & Sommer, 2012), benefitting from small size and concomitant lower nitrogen and phosphorus requirements, and a wide thermal range (Demory et al., 2019; Li et al., 2009). Here, we took on the eukaryotic picoplankton knowledge gap to extract functional insights from changes in *M. commoda*'s transcription patterns during growth with heterotrophic bacteria from three dominant marine bacterial classes.

The expression changes observed in *M. commoda* after only 8 h in bacterial co‐culture involved as much as 16% of its genes. This rapid and substantial transcriptome remodelling indicates a surprisingly extensive suite of metabolic and ecological connections between marine microbes. *M. commoda* bacterial recognition mechanisms allowed it to invoke different interactions for each associated bacterium, although the basis of this recognition was not readily decipherable from transcription patterns. Transcriptional responses by *M. commoda* were linked to the light‐dependent reactions of photosynthesis, CO_2_ fixation, central carbon metabolism, fatty acid biosynthesis, cell cycle control, biotin biosynthesis and nitrogen acquisition. Many of the responses differ from what has been characterised in co‐culture studies with other phytoplankton (Amin et al., 2015; Durham et al., 2017), emphasising the highly complex matrix of phytoplankton‐bacteria interactions that were not possible to imagine in the early history of marine microbial ecology (Cole, 1982).

## AUTHOR CONTRIBUTIONS

**Maria Hamilton:** Formal analysis (lead); investigation (supporting); visualization (lead); writing – original draft (lead); writing – review and editing (equal). **Frank Xavier Ferrer‐González:** Formal analysis (equal); investigation (supporting); methodology (equal); writing – review and editing (supporting). **Mary Ann Moran:** Funding acquisition (lead); methodology (equal); visualization (supporting); writing – review and editing (lead).

## CONFLICT OF INTEREST STATEMENT

The authors declare no conflict of interest.

## Supporting information


**Figure S1.** *M. commoda* genes with a significant shared response across all three bacterial co‐cultures categorised by KOG class. Shared response genes displayed in Figure 2 are not included here. A) Shared genes enriched in the co‐culture. B) Shared genes depleted in the co‐cultures. White asterisks indicate KOG categories with significantly higher numbers of genes that are different between the co‐culture enriched and depleted (exact binomial test, p‐value <0.05).
**Figure S2.** Pathway completeness for functions identified via KEGG‐decoder in the heterotrophic bacterial genomes. Completeness of each pathway from 0 (not present) to 1 (full pathway present) is indicated by the colour gradient. Functions not present in any of the three bacterial genomes are not displayed.

**Table S1.** Components of growth medium trace element and vitamin solutions.

**Table S2.** Modified and expanded manually curated functional gene annotations for *Micromonas commoda* RCC299.

## Data Availability

Raw data from this experiment are deposited in the NCBI SRA BioProject database under accession PRJNA787291. Data files are available on Zenodo: https://zenodo.org/doi/10.5281/zenodo.5822680 (Ferrer‐González et al., 2023).

## References

[emi413285-bib-0001] Amin, S.A. , Hmelo, L.R. , Van Tol, H.M. , Durham, B.P. , Carlson, L.T. , Heal, K.R. et al. (2015) Interaction and signalling between a cosmopolitan phytoplankton and associated bacteria. Nature, 522, 98–101. 26017307 10.1038/nature14488

[emi413285-bib-0002] Anders, S. , Pyl, P.T. & Huber, W. (2015) HTSeq‐A python framework to work with high‐throughput sequencing data. Bioinformatics, 31, 166–169. 25260700 10.1093/bioinformatics/btu638 PMC4287950

[emi413285-bib-0003] Armbrust, E.V. (2009) The life of diatoms in the world's oceans. Nature, 459, 185–192. 19444204 10.1038/nature08057

[emi413285-bib-0004] Armbrust, E.V. , Berges, J.A. , Bowler, C. , Green, B.R. , Martinez, D. , Putnam, N.H. et al. (2004) The genome of the diatom *Thalassiosira pseudonana*: ecology, evolution, and metabolism. Science, 306, 79–86. 15459382 10.1126/science.1101156

[emi413285-bib-0005] Armbrust, E.V. , Parker, M.S. , Rocap, G. , Jenkins, B. & Bates, S. (2011) Pseudo‐nitzschia multiseries CLN‐47 draft genome assembly. https://mycocosm.jgi.doe.gov/Psemu1/Psemu1.home.html

[emi413285-bib-0006] Atkins, K.C. & Cross, F.R. (2018) Interregulation of CDKA/CDK1 and the plant‐specific cyclin‐dependent kinase CDKB in control of the *Chlamydomonas* cell cycle. Plant Cell, 30, 429–446. 29367304 10.1105/tpc.17.00759 PMC5868683

[emi413285-bib-0007] Balch, W.M. , Drapeau, D.T. , Poulton, N. , Archer, S.D. , Cartisano, C. , Burnell, C. et al. (2023) Osmotrophy of dissolved organic compounds by coccolithophore populations: fixation into particulate organic and inorganic carbon. Scientific Advances, 9, eadf6973. 10.1126/sciadv.adf6973 PMC10208565 37224255

[emi413285-bib-0008] Balzano, S. , Corre, E. , Decelle, J. , Sierra, R. , Wincker, P. , Da Silva, C. et al. (2015) Transcriptome analyses to investigate symbiotic relationships between marine protists. Frontiers in Microbiology, 6, 1–14. 25852650 10.3389/fmicb.2015.00098 PMC4362344

[emi413285-bib-0009] Barak‐Gavish, N. , Dassa, B. , Kuhlisch, C. , Nussbaum, I. , Brandis, A. , Rosenberg, G. et al. (2023) Bacterial lifestyle switch in response to algal metabolites. eLife, 12, e84400. 36691727 10.7554/eLife.84400 PMC9873259

[emi413285-bib-0010] Bartolek, Z. , Van Creveld, S.G. , Coesel, S. , Cain, K.R. , Schatz, M. , Morales, R. et al. (2022) Flavobacterial exudates disrupt cell cycle progression and metabolism of the diatom *Thalassiosira pseudonana* . The ISME Journal, 16, 2741–2751. 36104452 10.1038/s41396-022-01313-9 PMC9666458

[emi413285-bib-0011] Bellande, K. , Bono, J.‐J. , Savelli, B. , Jamet, E. & Canut, H. (2017) Plant lectins and lectin receptor‐like kinases: how do they sense the outside? International Journal of Molecular Sciences, 18, 1164. 28561754 10.3390/ijms18061164 PMC5485988

[emi413285-bib-0012] Bender, S.J. , Durkin, C.A. , Berthiaume, C.T. , Morales, R.L. & Armbrust, E.V. (2014) Transcriptional responses of three model diatoms to nitrate limitation of growth. Frontiers in Marine Science, 1, 1–15.

[emi413285-bib-0013] Bowler, C. , Vardi, A. & Allen, A.E. (2010) Oceanographic and biogeochemical insights from diatom genomes. Annual Reviews Marine Science, 2, 333–365. 10.1146/annurev-marine-120308-081051 21141668

[emi413285-bib-0014] Bratbak, G. & Thingstad, T.F. (1986) Phytoplankton‐bacteria interactions: an apparent paradox? Analysis of a model system with both competition and commensalism. Deep Sea Research Part I Oceanographic Literature Review, 33, 236.

[emi413285-bib-0015] Calfee, B.C. , Glasgo, L.D. & Zinser, E.R. (2022) Prochlorococcus exudate stimulates heterotrophic bacterial competition with rival phytoplankton for available nitrogen. MBio, 13, e02571‐21. 35012332 10.1128/mbio.02571-21 PMC8749424

[emi413285-bib-0016] Clesceri, L. , Greenberg, A. & Eaton, A. (1998) Standard methods for the examination of water and wastewater, 20th edition. Washington, DC: APHA American Public Health Association.

[emi413285-bib-0017] Cole, J.J. (1982) Interactions between bacteria and algae in aquatic ecosystems. Annual Review of Ecology and Systematics, 13, 291–314.

[emi413285-bib-0018] Cooper, B. , Clarke, J.D. , Budworth, P. , Kreps, J. , Hutchison, D. , Park, S. et al. (2003) A network of rice genes associated with stress response and seed development. Proceedings of the National Academy of Sciences USA, 100, 4945–4950. 10.1073/pnas.0737574100 PMC153660 12684538

[emi413285-bib-0019] Cooper, M.B. , Kazamia, E. , Helliwell, K.E. , Kudahl, U.J. , Sayer, A. , Wheeler, G.L. et al. (2019) Cross‐exchange of B‐vitamins underpins a mutualistic interaction between *Ostreococcus tauri* and *Dinoroseobacter shibae* . The ISME Journal, 13, 334–345. 30228381 10.1038/s41396-018-0274-y PMC6331578

[emi413285-bib-0020] Croft, M.T. , Lawrence, A.D. , Raux‐Deery, E. , Warren, M.J. & Smith, A.G. (2005) Algae acquire vitamin B12 through a symbiotic relationship with bacteria. Nature, 438, 90–93. 16267554 10.1038/nature04056

[emi413285-bib-0021] Cuvelier, M.L. , Guo, J. , Ortiz, A.C. , Van Baren, M.J. , Tariq, M.A. , Partensky, F. et al. (2017) Responses of the picoprasinophyte *Micromonas commoda* to light and ultraviolet stress. PLoS One, 12, 1–27. 10.1371/journal.pone.0172135 PMC5344333 28278262

[emi413285-bib-0022] De Smet, I. , Voß, U. , Lau, S. , Wilson, M. , Shao, N. , Timme, R.E. et al. (2011) Unraveling the evolution of auxin signaling. Plant Physiology, 155, 209–221. 21081694 10.1104/pp.110.168161 PMC3075796

[emi413285-bib-0023] Demory, D. , Baudoux, A.C. , Monier, A. , Simon, N. , Six, C. , Ge, P. et al. (2019) Picoeukaryotes of the *micromonas* genus: sentinels of a warming ocean. The ISME Journal, 13, 132–146. 30116039 10.1038/s41396-018-0248-0 PMC6299001

[emi413285-bib-0024] Deng, Y. , Mauri, M. , Vallet, M. , Staudinger, M. , Allen, R.J. & Pohnert, G. (2022) Dynamic diatom‐bacteria consortia in synthetic plankton communities. Applied Environmental Microbiology, 88, e01619‐22. 36300970 10.1128/aem.01619-22 PMC9680611

[emi413285-bib-0025] Diaz, B.P. , Zelzion, E. , Halsey, K. , Gaube, P. , Behrenfeld, M. & Bidle, K.D. (2023) Marine phytoplankton downregulate core photosynthesis and carbon storage genes upon rapid mixed layer shallowing. The ISME Journal, 17, 1074–1088. 37156837 10.1038/s41396-023-01416-x PMC10284824

[emi413285-bib-0026] Diévart, A. & Clark, S.E. (2004) LRR‐containing receptors regulating plant development and defense. Development, 131, 251–261. 14701679 10.1242/dev.00998

[emi413285-bib-0027] Dow, L. (2021) How do quorum‐sensing signals mediate algae–bacteria interactions? Microorganisms, 9, 1391. 34199114 10.3390/microorganisms9071391 PMC8307130

[emi413285-bib-0028] Durham, B.P. , Dearth, S.P. , Sharma, S. , Amin, S.A. , Smith, C.B. , Campagna, S.R. et al. (2017) Recognition cascade and metabolite transfer in a marine bacteria‐phytoplankton model system. Environmental Microbiology, 19, 3500–3513. 28631440 10.1111/1462-2920.13834

[emi413285-bib-0029] Ferrer‐González, F.X. , Hamilton, M. , Smith, C.B. , Schreier, J.E. , Olofsson, M. & Moran, M.A. (2023) Bacterial transcriptional response to labile exometabolites from photosynthetic picoeukaryote *Micromonas commoda* . ISME Communications, 3, 1–11. 36690682 10.1038/s43705-023-00212-0 PMC9870897

[emi413285-bib-0030] Ferrer‐González, F.X. , Widner, B. , Holderman, N.R. , Glushka, J. , Edison, A.S. , Kujawinski, E.B. et al. (2021) Resource partitioning of phytoplankton metabolites that support bacterial heterotrophy. The ISME Journal, 15, 762–773. 33097854 10.1038/s41396-020-00811-y PMC8027193

[emi413285-bib-0031] Findlay, J.A. & Patil, A.D. (1984) Antibacterial constituents of the diatom *Navicula delognei* . Journal of Natural Products, 47, 815–818. 6512534 10.1021/np50035a010

[emi413285-bib-0032] González, J.M. , Fernández‐Gómez, B. , Fernàndez‐Guerra, A. , Gómez‐Consarnau, L. , Sánchez, O. , Coll‐Lladó, M. et al. (2008) Genome analysis of the proteorhodopsin‐containing marine bacterium *Polaribacter* sp. MED152 (flavobacteria). Proceedings of the National Academy of Sciences USA, 105, 8724–8729. 10.1073/pnas.0712027105 PMC2438413 18552178

[emi413285-bib-0033] Graham, E.D. , Heidelberg, J.F. & Tully, B.J. (2018) Potential for primary productivity in a globally‐distributed bacterial phototroph. The ISME Journal, 12, 1861–1866. 29523891 10.1038/s41396-018-0091-3 PMC6018677

[emi413285-bib-0034] Grigoriev, I.V. , Hayes, R.D. , Calhoun, S. , Kamel, B. , Wang, A. , Ahrendt, S. et al. (2021) PhycoCosm, a comparative algal genomics resource. Nucleic Acids Research, 49, D1004–D1011. 33104790 10.1093/nar/gkaa898 PMC7779022

[emi413285-bib-0035] Higgins, J.D. , Armstrong, S.J. , Franklin, F.C.H. & Jones, G.H. (2004) The *Arabidopsis MutS* homolog *AtMSH4* functions at an early step in recombination: evidence for two classes of recombination in *Arabidopsis* . Genes and Development, 18, 2557–2570. 15489296 10.1101/gad.317504 PMC529542

[emi413285-bib-0036] Jian, J. , Zeng, D. , Wei, W. , Lin, H. , Li, P. & Liu, W. (2017) The combination of RNA and protein profiling reveals the response to nitrogen depletion in *Thalassiosira pseudonana* . Scienctific Reports, 7, 8989. 10.1038/s41598-017-09546-x PMC5566445 28827639

[emi413285-bib-0037] Jimenez, V. , Burns, J.A. , Le Gall, F. , Not, F. & Vaulot, D. (2021) No evidence of phago‐mixotropy in *Micromonas polaris* (Mamiellophyceae), the dominant picophytoplankton species in the arctic. Journal of Phycology, 57, 433–446. 10.1111/jpy.13125 33394518

[emi413285-bib-0038] Kanehisa, M. , Sato, Y. , Kawashima, M. , Furumichi, M. & Tanabe, M. (2016) KEGG as a reference resource for gene and protein annotation. Nucleic Acids Research, 44, D457–D462. 26476454 10.1093/nar/gkv1070 PMC4702792

[emi413285-bib-0039] Kanehisa, M. , Sato, Y. & Morishima, K. (2016) BlastKOALA and GhostKOALA: KEGG tools for functional characterization of genome and metagenome sequences. Journal of Molecular Biology, 428, 726–731. 26585406 10.1016/j.jmb.2015.11.006

[emi413285-bib-0040] Kazamia, E. , Czesnick, H. , Nguyen, T.T.V. , Croft, M.T. , Sherwood, E. , Sasso, S. et al. (2012) Mutualistic interactions between vitamin B12‐dependent algae and heterotrophic bacteria exhibit regulation: algal‐bacterial interactions for delivery of vitamin B12. Environmental Microbiology, 14, 1466–1476. 22463064 10.1111/j.1462-2920.2012.02733.x

[emi413285-bib-0041] Kim, I. & Kim, H.U. (2022) The mysterious role of fibrillin in plastid metabolism: current advances in understanding. Journal of Experimental Botany, 73, 2751–2764. 35560204 10.1093/jxb/erac087

[emi413285-bib-0042] Kogure, K. , Simidu, U. & Taga, N. (1981) Bacterial attachment to phytoplankton in sea water. Journal of Experimental Marine Biology and Ecology, 56, 197–204.

[emi413285-bib-0043] Koonin, E.V. , Fedorova, N.D. , Jackson, J.D. , Jacobs, A.R. , Krylov, D.M. , Makarova, K.S. et al. (2004) A comprehensive evolutionary classification of proteins encoded in complete eukaryotic genomes. Genome Biology, 5, R7. 14759257 10.1186/gb-2004-5-2-r7 PMC395751

[emi413285-bib-0044] Landa, M. , Burns, A.S. , Roth, S.J. & Moran, M.A. (2017) Bacterial transcriptome remodeling during sequential co‐culture with a marine dinoflagellate and diatom. The ISME Journal, 11, 2677–2690. 28731474 10.1038/ismej.2017.117 PMC5702724

[emi413285-bib-0045] Langmead, B. & Salzberg, S.L. (2012) Fast gapped‐read alignment with bowtie 2. Nature Methods, 9, 357–359. 22388286 10.1038/nmeth.1923 PMC3322381

[emi413285-bib-0046] Le Reun, N. , Bramucci, A. , Ajani, P. , Khalil, A. , Raina, J.‐B. & Seymour, J.R. (2023) Temporal variability in the growth‐enhancing effects of different bacteria within the microbiome of the diatom *Actinocyclus sp* . Frontiers in Microbiology, 14, 1230349. 37608955 10.3389/fmicb.2023.1230349 PMC10440540

[emi413285-bib-0047] Li, W.K.W. , Mclaughlin, F.A. , Lovejoy, C. & Carmack, E.C. (2009) Smallest algae thrive as the arctic. Science, 326, 539. 19900890 10.1126/science.1179798

[emi413285-bib-0048] Ligterink, W. & Hirt, H. (2001) Mitogen‐activated protein (MAP) kinase pathways in plants: versatile signaling tools. International Review of Cytology, 201, 209–275. 11057833 10.1016/s0074-7696(01)01004-x

[emi413285-bib-0049] Longnecker, K. , Kido Soule, M.C. , Swarr, G.J. , Parsons, R.J. , Liu, S. , Johnson, W.M. et al. (2024) Seasonal and daily patterns in known dissolved metabolites in the northwestern Sargasso Sea. Limnology & Oceanography, 9999, 1–18.

[emi413285-bib-0050] Love, M.I. , Huber, W. & Anders, S. (2014) Moderated estimation of fold change and dispersion for RNA‐seq data with DESeq2. Genome Biology, 15, 1–21. 10.1186/s13059-014-0550-8 PMC4302049 25516281

[emi413285-bib-0051] Lovejoy, C. , Massana, R. & Pedro, C. (2006) Diversity and distribution of marine microbial eukaryotes in the arctic ocean and adjacent seas diversity and distribution of marine microbial eukaryotes in the arctic ocean and adjacent seas. Applied and Environmental Microbiology, 72, 3085–3095. 16672445 10.1128/AEM.72.5.3085-3095.2006 PMC1472370

[emi413285-bib-0052] Massana, R. (2011) Eukaryotic picoplankton in surface oceans. Annual Review of Microbiology, 65, 91–110. 10.1146/annurev-micro-090110-102903 21639789

[emi413285-bib-0053] Mayali, X. , Franks, P. & Burton, R. (2011) Temporal attachment dynamics by distinct bacterial taxa during a dinoflagellate bloom. Aquatic Microbial Ecology, 63, 111–122.

[emi413285-bib-0054] McCarren, J. , Becker, J.W. , Repeta, D.J. , Shi, Y. , Young, C.R. , Malmstrom, R.R. et al. (2010) Microbial community transcriptomes reveal microbes and metabolic pathways associated with dissolved organic matter turnover in the sea. Proceedings of the National Academy of Sciences USA, 107, 16420–16427. 10.1073/pnas.1010732107 PMC2944720 20807744

[emi413285-bib-0055] McDonald, S.M. , Plant, J.N. & Worden, A.Z. (2010) The mixed lineage nature of nitrogen transport and assimilation in marine eukaryotic phytoplankton: a case study of *micromonas* . Molecular Biology and Evolution, 27, 2268–2283. 20457585 10.1093/molbev/msq113 PMC2944026

[emi413285-bib-0056] Miller, R. , Wu, G. , Deshpande, R.R. , Vieler, A. , Gartner, K. , Li, X. et al. (2010) Changes in transcript abundance in *Chlamydomonas reinhardtii* following nitrogen deprivation predict diversion of metabolism. Plant Physiology, 154, 1737–1752. 20935180 10.1104/pp.110.165159 PMC2996024

[emi413285-bib-0057] Millette, N.C. , Gast, R.J. , Luo, J.Y. , Moeller, H.V. , Stamieszkin, K. , Andersen, K.H. et al. (2023) Mixoplankton and mixotrophy: future research priorities. Journal of Plankton Research, 45, 576–596. 37483910 10.1093/plankt/fbad020 PMC10361813

[emi413285-bib-0058] Moran, M.A. , Ferrer‐González, F.X. , Fu, H. , Nowinski, B. , Olofsson, M. , Powers, M.A. et al. (2022) The Ocean's labile DOC supply chain. Limnology and Oceanography, 67, 1–15.

[emi413285-bib-0059] Morán, X.A.G. , López‐Urrutia, Á. , Calvo‐Díaz, A. & Li, W.K.W. (2010) Increasing importance of small phytoplankton in a warmer ocean. Global Change Biology, 16, 1137–1144.

[emi413285-bib-0060] Muñoz‐Marín, M.D.C. , Luque, I. , Zubkov, M.V. , Hill, P.G. , Diez, J. & García‐Fernández, J.M. (2013) *Prochlorococcus* can use the Pro1404 transporter to take up glucose at nanomolar concentrations in the Atlantic Ocean. Proceedings of the National Academy of Sciences USA, 110, 8597–8602. 10.1073/pnas.1221775110 PMC3666668 23569224

[emi413285-bib-0061] Nymark, M. , Valle, K.C. , Brembu, T. , Hancke, K. , Winge, P. , Andresen, K. et al. (2009) An integrated analysis of molecular acclimation to high light in the marine diatom *Phaeodactylum tricornutum* . PLoS One, 4, e7743. 19888450 10.1371/journal.pone.0007743 PMC2766053

[emi413285-bib-0062] Polovina, J.J. , Howell, E.a. & Abecassis, M. (2008) Ocean's least productive waters are expanding. Geophysical Research Letters, 35, 2–6.

[emi413285-bib-0063] Poulsen, N. , Chesley, P.M. & Kröger, N. (2006) Molecular genetic manipulation of the diatom *Thalassiosira pseudonana* (Bacillariophyceae). Journal of Phycology, 42, 1059–1065.

[emi413285-bib-0064] Segev, E. , Wyche, T.P. , Kim, K.H. , Petersen, J. , Ellebrandt, C. , Vlamakis, H. et al. (2016) Dynamic metabolic exchange governs a marine algal‐bacterial interaction. eLife, 5, e17473. 27855786 10.7554/eLife.17473 PMC5148602

[emi413285-bib-0065] Seyedsayamdost, M.R. , Case, R.J. , Kolter, R. & Clardy, J. (2011) The Jekyll‐and‐Hyde chemistry of *Phaeobacter gallaeciensis* . Nature Chemistry, 3, 331–335. 10.1038/nchem.1002 PMC3376411 21430694

[emi413285-bib-0066] Seymour, J.R. , Amin, S.A. , Raina, J.B. & Stocker, R. (2017) Zooming in on the phycosphere: the ecological interface for phytoplankton‐bacteria relationships. Nature Microbiology, 2, 17065. 10.1038/nmicrobiol.2017.65 28555622

[emi413285-bib-0067] Sharma, A.K. , Becker, J.W. , Ottesen, E.A. , Bryant, J.A. , Duhamel, S. , Karl, D.M. et al. (2014) Distinct dissolved organic matter sources induce rapid transcriptional responses in coexisting populations of *Prochlorococcus*, *Pelagibacter* and the OM60 clade. Environmental Microbiology, 16, 2815–2830. 24118765 10.1111/1462-2920.12254

[emi413285-bib-0068] Sibbald, S.J. & Archibald, J.M. (2017) More protist genomes needed. Nature Ecology and Evolution, 1, 145. 28812681 10.1038/s41559-017-0145

[emi413285-bib-0069] Singh, D.K. , Maximova, S.N. , Jensen, P.J. , Lehman, B.L. , Ngugi, H.K. & McNellis, T.W. (2010) *FIBRILLIN4* is required for plastoglobule development and stress resistance in apple and *Arabidopsis* . Plant Physiology, 154, 1281–1293. 20813909 10.1104/pp.110.164095 PMC2971606

[emi413285-bib-0070] Spaepen, S. & Vanderleyden, J. (2011) Auxin and plant‐microbe interactions. Cold Spring Harbor Perspectives in Biology, 3, a001438. 21084388 10.1101/cshperspect.a001438 PMC3062209

[emi413285-bib-0071] Stocker, R. & Seymour, J.R. (2012) Ecology and physics of bacterial chemotaxis in the ocean. Microbiology and Molecular Biology Reviews, 76, 792–812. 23204367 10.1128/MMBR.00029-12 PMC3510523

[emi413285-bib-0072] Sun, D. , Zhu, J. , Fang, L. , Zhang, X. , Chow, Y. & Liu, J. (2013) *De novo* transcriptome profiling uncovers a drastic downregulation of photosynthesis upon nitrogen deprivation in the nonmodel green alga *Botryosphaerella sudeticus* . BMC Genomics, 14, 715. 24138407 10.1186/1471-2164-14-715 PMC4050207

[emi413285-bib-0073] Tzvetkova‐Chevolleau, T. , Franck, F. , Alawady, A.E. , Dall'Osto, L. , Carrière, F. , Bassi, R. et al. (2007) The light stress‐induced protein ELIP2 is a regulator of chlorophyll synthesis in *Arabidopsis thaliana*: ELIP and chlorophyll synthesis. The Plant Journal, 50, 795–809. 17553115 10.1111/j.1365-313X.2007.03090.x

[emi413285-bib-0074] van Baren, M.J. , Bachy, C. , Reistetter, E.N. , Purvine, S.O. , Grimwood, J. , Sudek, S. et al. (2016) Evidence‐based green algal genomics reveals marine diversity and ancestral characteristics of land plants. BMC Genomics, 17, 267. 27029936 10.1186/s12864-016-2585-6 PMC4815162

[emi413285-bib-0075] Ware, M.A. , Giovagnetti, V. , Belgio, E. & Ruban, A.V. (2015) PsbS protein modulates non‐photochemical chlorophyll fluorescence quenching in membranes depleted of photosystems. Journal of Photochemistry and Photobiology B: Biology, 152, 301–307. 26233261 10.1016/j.jphotobiol.2015.07.016

[emi413285-bib-0076] Widner, B. , Kido Soule, M.C. , Ferrer‐González, F.X. , Moran, M.A. & Kujawinski, E.B. (2021) Quantification of amine‐ and alcohol‐containing metabolites in saline samples using pre‐extraction benzoyl chloride derivatization and ultrahigh performance liquid chromatography tandem mass spectrometry (UHPLC MS/MS). Analytical Chemistry, 93, 4809–4817. 33689314 10.1021/acs.analchem.0c03769

[emi413285-bib-0077] Wilken, S. , Yung, C.C.M. , Hamilton, M. , Hoadley, K. , Nzongo, J. , Eckmann, C. et al. (2019) The need to account for cell biology in characterizing predatory mixotrophs in aquatic environments. Philosophical Transactions of the Royal Society B: Biological Sciences, 374, 20190090. 10.1098/rstb.2019.0090 PMC6792458 31587652

[emi413285-bib-0078] Winder, M. & Sommer, U. (2012) Phytoplankton response to a changing climate. Hydrobiologia, 698, 5–16.

[emi413285-bib-0079] Wood‐Charlson, E.M. , Hollingsworth, L.L. , Krupp, D.A. & Weis, V.M. (2006) Lectin/glycan interactions play a role in recognition in a coral/dinoflagellate symbiosis. Cellular Microbiology, 8, 1985–1993. 16879456 10.1111/j.1462-5822.2006.00765.x

[emi413285-bib-0080] Worden, A.Z. , Lee, J.H. , Mock, T. , Rouzé, P. , Simmons, M.P. , Aerts, A.L. et al. (2009) Green evolution and dynamic adaptions revealed by genomes of the marine picoeukaryotes *micromonas* . Science, 324, 268–272. 19359590 10.1126/science.1167222

[emi413285-bib-0081] Worden, A.Z. , Nolan, J.K. & Palenik, B. (2004) Assessing the dynamic and ecology of marine picoplankton : the importance of eukaryotic component. Limnology and Oceanography, 49, 168–179.

[emi413285-bib-0082] Yelton, A.P. , Acinas, S.G. , Sunagawa, S. , Bork, P. , Pedrós‐Alió, C. & Chisholm, S.W. (2016) Global genetic capacity for mixotrophy in marine picocyanobacteria. The ISME Journal, 10, 2946–2957. 27137127 10.1038/ismej.2016.64 PMC5148188

[emi413285-bib-0083] Yue, J. , Meyers, B.C. , Chen, J. , Tian, D. & Yang, S. (2012) Tracing the origin and evolutionary history of plant nucleotide‐binding site–leucine‐rich repeat (NBS‐LRR) genes. New Phytologist, 193, 1049–1063. 22212278 10.1111/j.1469-8137.2011.04006.x

[emi413285-bib-0084] Zhu, S.‐H. & Green, B.R. (2010) Photoprotection in the diatom *Thalassiosira pseudonana*: role of LI818‐like proteins in response to high light stress. Biochimica et Biophysica Acta (BBA)‐Bioenergetics, 1797, 1449–1457. 20388491 10.1016/j.bbabio.2010.04.003

