# Supplementary material for: Heterotrophic bacteria trigger transcriptome remodelling in the photosynthetic picoeukaryote Micromonas commoda
Source: Environ Microbiol Rep. 2024 May 22;16(3):e13285. doi: 10.1111/1758-2229.13285 (PMC11112143; doi:10.1111/1758-2229.13285)
Supplement: Supplementary file 1 — Figure S1. M. commoda genes with a significant shared response across all three bacterial co‐cultures categorised by KOG class. Shared response genes displayed in Figure 2 are not included here. A) Shared genes enriched in the co‐culture. B) Shared genes depleted in the co‐cultures. White asterisks indicate KOG categories with significantly higher numbers of genes that are different between the co‐culture enriched and depleted (exact binomial test, p‐value <0.05). Figure S2. Pathway completeness for functions identified via KEGG‐decoder in the heterotrophic bacterial genomes. Completeness of each pathway from 0 (not present) to 1 (full pathway present) is indicated by the colour gradient. Functions not present in any of the three bacterial genomes are not displayed. [file EMI4-16-e13285-s002.pdf]

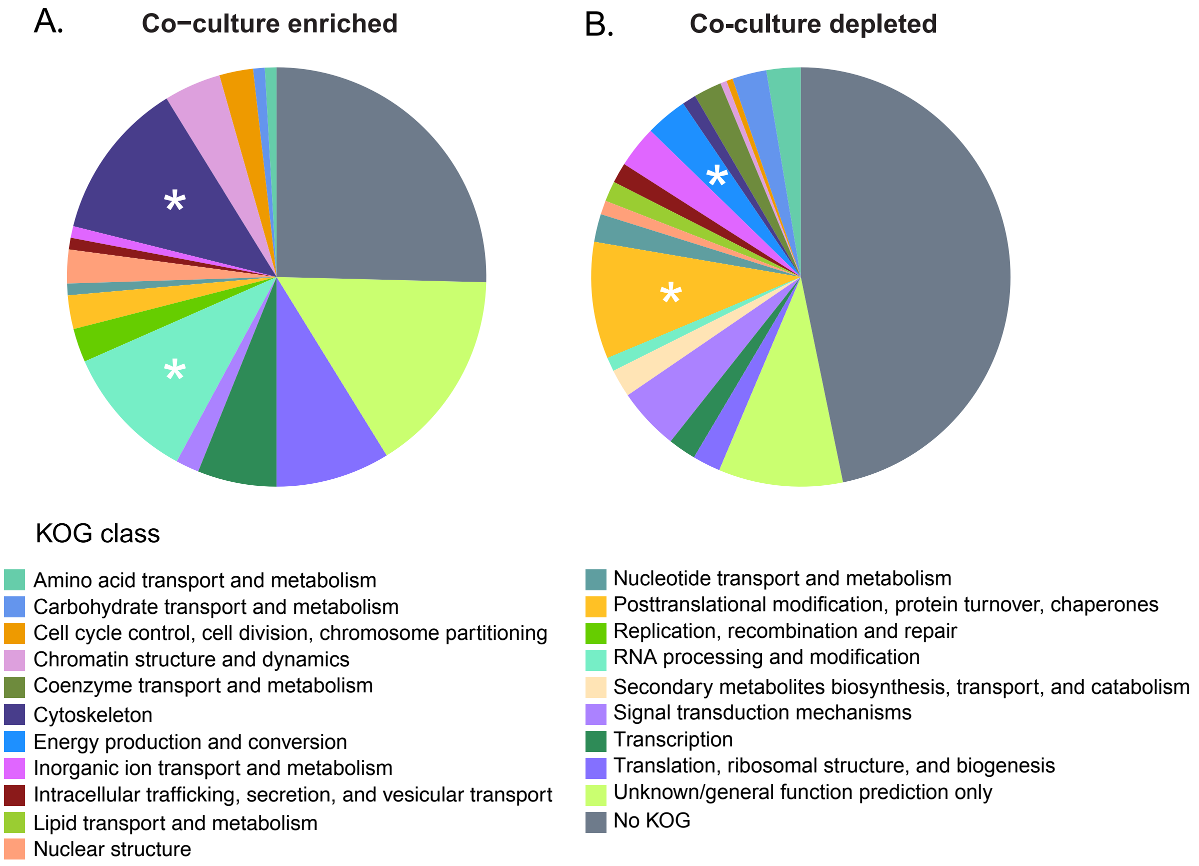

Supplemental Figure 1

M. commoda genes with a significant shared response across all three bacterial co-cultures categorized by KOG class. Shared response genes displayed in Figure 2 are not included here. A) Shared genes enriched in the co-culture. B) Shared genes depleted in the co-cultures. White asterisks indicate KOG categories with significantly higher numbers of genes that are different between the co-culture enriched and depleted (exact binomial test, p-value ≤ 0.05).

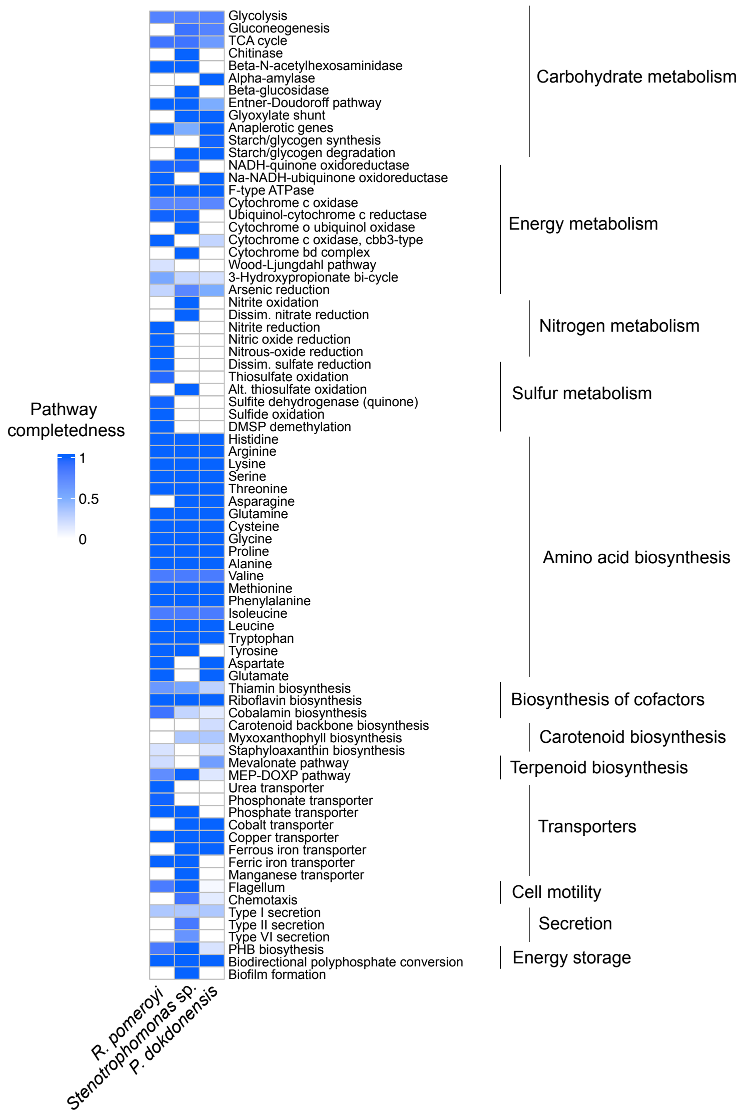

Supplemental Figure 2

Pathway completeness for functions identified via KEGG-decoder in the heterotrophic bacterial genomes. Completeness of each pathway from 0 (not present) to 1 (full pathway present) is indicated by the color gradient. Functions not present in any of the three bacterial genomes are not displayed.
