# Supplementary material for: Heterotrophic bacteria trigger transcriptome remodelling in the photosynthetic picoeukaryote Micromonas commoda
Source: Environ Microbiol Rep. 2024 May 22;16(3):e13285. doi: 10.1111/1758-2229.13285 (PMC11112143; doi:10.1111/1758-2229.13285)
Supplement: Supplementary file 2 — Table S1. Components of growth medium trace element and vitamin solutions. [file EMI4-16-e13285-s003.docx]

| **Table S1. Components of growth medium trace element and vitamin solutions.** | |
| --- | --- |
| **L1 Trace Element Solution** | |
| **Component** | **Molar concentration in final medium** |
| Na_2_EDTA · 2H_2_O | 1.17 x 10-5 M |
| FeCl_3_ · 6H_2_O | 1.17 x 10^-5^ M |
| MnCl_2_·4 H_2_O | 9.09 x 10^-7^ M |
| ZnSO_4_ · 7H_2_O | 8.00 x 10^-8^ M |
| CoCl_2_ · 6H_2_O | 5.00 x 10^-8^ M |
| CuSO_4_ · 5H_2_O | 1.00 x 10^-8^ M |
| Na_2_MoO_4_ · 2H_2_O | 8.22 x 10^-8^ M |
| H2SeO_3_ | 1.00 x 10^-8^ M |
| NiSO_4_ · 6H_2_O | 1.00 x 10^-8^ M |
| Na_3_VO_4_ | 1.00 x 10^-8^ M |
| K_2_CrO | 1.00 x 10^-8^ M |
| **L1 Vitamin Solution** | |
| **Component** | **Molar concentration in final medium** |
| thiamine · HCl (vit. B_1_) | 2.96 x 10^-7^ M |
| biotin (vit. B7) | 2.05 x 10^-9^ M |
| cyanocobalamin (vit. B_12_) | 3.69 x 10^-10^ M |
